# 

**Published:** 1894-07

**Authors:** 


					﻿THE
Southern Medical Record.
A Monthly Journal of Medicine and Surgery.
Vol. XXIV.	ATLANTA, GA., JULY, 1894.	No. 7.
EDITORS :
A. W. GRIGGS, M. D.
j. McFadden Gaston, m. d.
WILLIS. F. WESTMORELAND, M.T>.
LOUIS H. JONES, M. D.
DAN. H. HOWELL, M. D., Business Manager.
Entered at the Postoftice at Atlanta, Ga., as Second-Class Matter.
The Franklin Printing and Publishing Co., Atlanta, Ga.
				

